# Factors associated with dietary diversity among adolescents in Woldia, Northeast Ethiopia

**DOI:** 10.1186/s40795-021-00430-6

**Published:** 2021-04-29

**Authors:** Melese Linger Endalifer, Gashaw Andargie, Bekri Mohammed, Bedilu Linger Endalifer

**Affiliations:** 1 Department of Public Health, College of Health Science, Woldia University, Woldia, Ethiopia; 2 Institute of Public Health, College of Medicine and Health Science, University of Gondar, Gondar, Ethiopia; 3 Department of Pharmacy, College of Medicine and Health Science, Wollo University, Dessie, Ethiopia

**Keywords:** Adolescent, Dietary diversity, Woldia

## Abstract

**Background:**

Consuming diversified food during the adolescent period is essential to build a healthy and active mind for their later life. Food prices increased in the local market due to fewer production of crops. Thus, exploring the dietary diversity of adolescents in this area is crucial to estimate diet quality. So the aim of the study was to identify determinant factors of dietary diversity.

**Methods:**

An institution-based cross-sectional study was conducted among adolescent students in Woldia town. A total of four hundred eleven students were included in the study. A simple random sampling technique was used to select the participants. The outcome variable was dietary diversity; it was calculated by summing of the number of food group consumed by individuals in the given reference period. Bivariable and multivariable logistic analysis was done. The odds ratio with a 95% confidence interval was computed to measure an association. A variable with a *P*-value less than 0.05 is considered a significant factor.

**Results:**

The proportion of inadequate dietary diversity was 49.1% (95% CI 44.5–53.8). Being female (AOR =5.53, 95% CI 3.447–8.859), secondary and above mothers’ education level (AOR=0. 27, 95%CI 0.153–0.477), living in a family size five and above (AOR= 2.09, 95CI% 1.31–3.34), and poor knowledge about nutrition (AOR=4.56, 95% CI 2.727–7.639) were significantly associated with inadequate dietary diversity.

**Conclusions and recommendations:**

Inadequate dietary diversity was associated with sex, knowledge of nutrition, maternal education level, and family size. It is better to design a nutrition intervention program that focus on nutrition education to scale up diversified food consumption among adolescents.

**Supplementary Information:**

The online version contains supplementary material available at 10.1186/s40795-021-00430-6.

## Background

Dietary diversity is defined as the simple count of food groups consumed over a given reference period [1]. Individual dietary diversity score is a good predictor for nutrient adequacy [2, 3] and nutritional status [4]. Dietary diversity is an indicator of a balanced diet and normal weight status [5]. Practically dietary diversity can be assessed at an individual or household level, whereas the dietary diversity assessed at the household level is a proxy indicator of food security or insecurity in the family [6].

The adolescent period is a critical age in which nutritional requirement is high due to fast growth and development. Healthy dietary practices during the adolescent period affect the latter life cycle either positively or negatively [7]. Eating habit of adolescents was influenced by multiple factors such as lifestyle, food marketing, media, socioeconomic, and cultural factors [8].

Adolescents who live in the community with low awareness and practice about healthy eating suffered more nutrition-related problems [9]. Besides, adolescents in developing countries do not get enough nutrients due to monotonous dietary dish [10].

In Ethiopia, different scholars intensively identified the determinant factors of dietary diversity among under-five children and pregnant and lactating women, but there is limited research regarding adolescent dietary practices [11–28]. Previous articles conducted in Ethiopia focus on the nutritional status of adolescent either they identify chronic deficiency or overweight/obesity. Dietary diversity was studied as a determinant factor of malnutrition previously in adolescents. But not the factors that influence dietary diversity was not well explored [29–37]. Furthermore, the dietary diversity of adolescents was not included and studied in Ethiopia Demographic and Health Survey consecutive reports [38–40].

In the majority of the developing countries, assessment of adolescent dietary diversity is not common. Data regarding adolescent nutrition was limited in Ethiopia [10, 41]. North Wollo Zone is part of Ethiopia which is frequently affected by drought; for instance, it was affected by ELINO in 2015/2016. Due to the ELINO drought, food prices were highly increased in Woldia town because of the reduction of crop production in the district around there [42, 43]. The World Health Organization (WHO) and the Food and Agricultural Organization (FAO) recommended dietary diversity assessment during drought season since it is important to know the diet quality of the population clearly [44]. Because of the aforementioned reason, we intend to assess the dietary diversity and its associated factors among adolescents in Woldia Secondary Schools.

## Materials and methods

### Study design and period

An institution-based cross-sectional study was conducted from February to March 2016 G.c. Woldia is one of the oldest towns in Ethiopia and the center of the North Wollo Zone. It is located 525 km North of Addis Ababa the capital city of Ethiopia and 360 km far from Bahir Dar the capital of Amhara Regional state.

Based on the 2007 National census conducted by the Central Statistical Agency of Ethiopia (CSA), the town has a total population of 46,139, of whom 23,000 are men and 23,139 women. The town has four secondary schools (one preparatory school (grades 11 and 12) and 3 general high schools (grade 9 and 10)). A total of 4298 students attend their education in the 2015/2016 academic year.

### Source population and eligibility criteria

All adolescent students who were attending their education in Woldia Secondary Schools in 2015/2016 were considered as source population. Those students who participated in different ceremonies and were ill before a day of the survey were excluded from the study.

Outcome variables: The dietary diversity score was an outcome variable. It was coded as “1” inadequate dietary diversity and “0” as adequate dietary diversity. The dichotomization was based on mean dietary diversity score. That is inadequate DDS (≤4.75) and adequate DDS (>4.75).

Independent variables: sociodemographic characteristics and socioeconomic and behavioral factors.

### Sample size determination and sampling technique

Single population proportion formula was done to estimate sample size with the following assumptions *N*= *Z*_α/2_^2^*p* (1 − *p*)/*d*^2^= (1.96)^2^*0.5*0.5/0.0025= 384.The assumption is *Z* = level of confidence (1.96), *p* = proportion of inadequate dietary diversity was taken as 50%, *d*=s margin of error (5%), *N*= sample size, and thorough consideration of 10% non-response rate the final sample size was 422. In Woldia town, there are four secondary schools namely, Selama, Millennium, Woldia General, and Woldia Preparatory Schools which were included as source population. In the four schools, 4298 students attend their education of which 4095 students were adolescents. Then, the student list was collected from the respective school directors and aggregated to one data set. Then the combined student data set list contains information about grade level, school name, section, and students’ identity number. Finally, through a simple random sampling technique, 422 students were selected through openepi random number computer generator.

### Data collection tools and procedures

Dietary Diversity Score (DDS) was computed using Food and Agriculture Organizations (FAO) 1-day individual dietary diversity questionnaire. Due to the lack of local food, dietary guidelines in Ethiopia food items were identified through market observation and by collecting hotel meal menus. Around fifty food items were identified and aggregated with the FAO food grouping system. Ten food groups were formed; the formed food groups were cereals, vitamin A-rich vegetables, white roots and tubers, dark green leafy vegetables, other vegetables and fruits, organ and flesh meat, eggs, legumes and seeds, milk and milk products, oils, and fats. To collect the necessary data, semi-structured interview was used. Food item eaten by an individual a day before the interview was recorded in a food group table .Finally, DDS was constructed by counting food groups consumed by adolescents over 24 h [45]. Additionally, sixteen nutritional knowledge questions were adapted from FAO guidelines to measure nutrition-related knowledge, attitudes, and practices [46]. Individuals who responded less than nine nutrition knowledge questions were considered as having poor nutrition knowledge and those who answered ≥9 questions were categorized as having good knowledge. The content of nutritional knowledge question was composed of the cause and prevention mechanism of (iodine deficiency, vitamin A deficiency, iron deficiency anemia, and protein energy malnutrition).

Five Bachelor of Science (BSc.) Nursing students were recruited for data collection. The principal investigator had taken written consent from their parents and was granted their consent for those students whose ages were below 18 years. The list of randomly selected students’ identity number was given to the data collectors. Data collectors introduce themselves and explain the purpose of the study to each study participant. At the end, the data were collected after taking verbal assent from each participant. Nutrition counseling was given for the students to practice diversified dietary habits.

### Data quality control

A pretest was performed before the actual data collection in Mersa Town. A 1-day training was given to the data collectors how they can conduct an interview. The questionnaire was initially prepared in English and translated to Amharic version. Double-entry of the data was done by two independent personnel.

### Data management and analysis

The data were entered into the Epi-Data version 3.1, cleaned manually, and exported to SPSS (Statistical Package for Social Science) version 20 for analysis. Descriptive statistics were presented using mean, median, standard deviations, interquartile range, and frequency table. Wealth index was analyzed through the principal component analysis (PCA) method and classified as (low, middle, and high).The bivariable and multivariable logistic regression analyses were performed. Finally, adjusted odds ratio with 95% CI was computed and variables having *P*-value less than 0.05 were considered as significant. The model was fitted with Hosmer and Lemeshow *P*-value of 0.89.

## Results

### Socio-demographic characteristics

A total of 411 adolescents were interviewed with a response rate of 97.3%. From 422 randomly selected participants, 11 students were excluded due to the following reasons: 4 students were refusing to participate in the study and 7 students were experiencing illness one day before the interview, which affect usual eating habit.

The median age of the students was 17 years (interquartile range 2) and 279 (67.2%) adolescents were aged 17–19 years. More than two thirds, 296 (72.0%), were Orthodox and 104 (25.3%) were Muslims in religion. Concerning the occupation of the parents, 251 (61.1%) of adolescents’ mothers were housewives and 272 (66.2%) of adolescent’s fathers were private workers.

Regarding the participants’ source of food, 304 (74.0%) got from the market. Fifty percent of adolescents live in a family size of five and above (Table 1).

**Table 1:** Socio-demographic characteristics of adolescents in Woldia Secondary Schools, Northeast Ethiopia, 2016 (*N*=411)

Variables	Category	Frequency (*n*)	Percentage (%)
Sex	Female	210	51.1
Age	14–16	135	32.8
	17–19	276	67.2
Residence	Urban	390	94.9
	Rural	21	5.1
Religion	Orthodox	296	72
	Muslim	104	25.3
	Other*	11	2.7
Grade level	9–10	270	65.7
	11–12	141	34.3
Maternal education	No formal education	147	35.8
	Elementary School	122	29.7
	Secondary and above	142	34.5
Paternal education	No formal education	112	27.3
	Elementary school	121	29.4
	Secondary and above	178	43.3
Occupation of fathers	Private worker	272	66.2
	Government	126	30.7
	Other**	13	3.2
Occupation of mothers	House wife	251	61.1
	Private worker	102	24.8
	Government employee	55	13.4
	Others**	3	0.7
Source of food	Market	304	74
	Farming	87	21.2
	Others***	20	4.9
Family size	<5	206	50.1
	≥5	205	49.9

*Protestant

**Unemployed

***Relatives

### Behavioral-related characteristics of adolescents

Among 411 participants, 282 (68.6%) had good knowledge of nutrition and 281 (68.4%) of them did not eat outside home in the last week. Moreover, more than half of them were satisfied with their current body weight and 283 (68.9%) ate their meals with their family members (Table 2).

**Table 2:** Behavioral-related characteristics of the adolescent in Woldia Secondary Schools, Northeast Ethiopia, 2016 (*N*=411)

Variables	Category	*N* (%)
Current bodyweight satisfaction	Wants to increase	106(25.8)
	Satisfied	226 (55)
	Wants to decrease	79 (19.2)
Eating out practice	None	281 (68.4)
	2–4per weak	99 (24.1)
	≥5 per week	31 (7.5)
Eating companions	With family members	283 (68.9)
	With peers	48 (11.7)
	Eats alone	80 (19.5)
Knowledge of nutrition	Good	282 (68.6)

### The magnitude of inadequate dietary diversity

The mean dietary diversity score was 4.73 (SD±1. 186) that ranged from 2 to 10 food groups. The proportion of inadequate dietary diversity among adolescents in Woldia Secondary Schools was 49.1% (95% CI 44.5–53.8).

Majority of the participants, 166 (40.4%), consumed four and 125 (30.4%) consumed five food groups computed from the total food category. More than 50% of the adolescent ate cereals, other vegetables and fruits, legumes, oils, and fat food groups. However, vitamin A-rich foods, milk, and eggs were consumed in a small proportion of the adolescents. Animal source food consumed by minor adolescents; 94 (22.9%) took flesh and organ meat, 41 (10.0 %) ate eggs, and 32 (7.8%) consumed milk and its products. Specifically eggs, milk, white root and tubers, green leafy vegetables, and vitamin A source fruits were almost not consumed by adolescents who had inadequate dietary diversity (Table 3).

**Table 3:** The proportion of food group consumption tabulated with dietary diversity among adolescents in Woldia Secondary Schools, Northeast Ethiopia, 2016 (*N*=411)

Food groups	YES ***N*** (%)	Adequate DDS ***N*** (%)	Inadequate DDS ***N*** (%)
Cereals	410 (99.8)	209 (50.9)	201 (48.9)
Vitamin A-rich fruits and vegetables	74 (18.0)	68 (16.5)	6 (1.5)
White roots and tubers	94 (22.9)	90 (21.9)	4 (1)
Dark green leafy vegetables	86 (20.9)	81 (19.7)	5 (1.2)
Other vegetables and fruits	390 (94.9)	208 (50.6)	182 (44.3)
Organ and flesh meat	94 (22.9)	68 (16.5)	26 (6.3)
Eggs	41 (10.0)	41 (10)	0 (0)
Milk and milk products	32 (7.8)	27 (6.6)	5 (1.2)
Legumes and nuts	343 (83.5)	181 (44)	162 (39.4)
Oils and fats	381 (92.7)	204 (49.6)	177 (43.1)
Mean dietary diversity	4.73		

### Factors associated with dietary diversity

All variables were entered into multivariable logistic regression model, and out of these, four variables had a significant association with inadequate dietary diversity.

Thus, age, residence, occupations of mother, religion, occupation of father, paternal education, source of food, wealth index, and current body weight satisfaction, eating out practice, and eating companions had no significant association with dietary diversity.

On the contrary, being female, the education level of adolescents’ mother, poor knowledge on nutrition, and living in five and above family size had a significant association with inadequate dietary diversity.

The odds of having inadequate dietary diversity among female was 5.526 times higher than male (AOR=5.53, 95% CI 3.447–8.859).The practice of inadequate dietary diversity was decreased by 73% among adolescents whose mother education level was secondary and above (AOR=0.27, 95% CI 0.153–0.477). The odds of having inadequate dietary diversity among adolescents living in a family size of five and above were 2.09 times higher than adolescents who lived in a family size less than five (AOR=2.092, 95 CI% 1.31–3.34).The odds of inadequate dietary diversity among adolescents who had poor knowledge of nutrition increased by 4.56 times than the adolescents who had good knowledge of nutrition (AOR=4.564, 95% CI 2.727–7.639) (Table 4).

**Table 4:** Multivariable logistic regression analysis of factors associated with inadequate dietary diversity among adolescents in Woldia Secondary Schools, Northeast Ethiopia, 2016 (*N*=411)

			Inadequate DDS *N* (%)	COR (95%CI)	AOR (95% CI)
**Sex**	Male*	201	60 (14.6)	1	1
	Female	210	142 (34.5)	4.91 (3.23–7.455)	5.53(3.447–8.859)
**Maternal education**	No formal education	147	90 (21.9)	1	1
	Elementary school	122	68 (16.5)	0.79(0.49–1.299)	0.78(0.45–1.365)
	Secondary school and above	142	44 (10.7)	0.28 (0.175–0.46)	0.27 (0.153–0.477)
**Family size**	<5	206	89 (21.7)	1	1
	≥ 5	205	113 (27.5)	1.61 (1.094–2.384)	2.09 (1.31–3.34)
**Nutritional knowledge**	Good	282	107 (26)	1	1
	Poor	129	95 (23.1)	4.57 (2.886–7.236)	4.56 (2.727–7.639)

*Reference category, Backward LR method was used

## Discussion

The overall mean dietary diversity score was 4.73 (SD ±1. 186) which ranged from 2 to 10 food groups. The proportion of inadequate dietary diversity among adolescents in Woldia Secondary Schools was 49.1 % (95% CI 44.5–53.8).

The proportion of inadequate dietary diversity among adolescents was 49.1% which is similar to the study carried out in the Amhara region among female adolescents [4], but it is smaller than a study conducted in Jimma 80.5% [47]. Thus, the variation might occur because of the reference period difference to calculate DDS, the number of food groups included in the score, and the study setting.

The current study is higher than a study conducted in Iran among female adolescents 21.3% [48]. The disparity might happen due to socio-economic differences and the presence of food-based dietary guidelines in Iran which promote diversified food consumption.

Meanwhile, the mean DDS of the current study was consistent with a study done in India 5.75 [49], Amhara Region among female adolescents 5.6 [4], Tigray region among female adolescents 3.5 [50], and Bangladesh among adult females 4.28 [51].

On the contrary, the mean DDS of this study was lower than a study conducted in Ahvaz 6.81 [48] and Tehran 6.25 [2]. Possibly the difference could be due to the variation of food groups included and socioeconomic differences among study participants.

In this study, cereals were consumed by all participants; this is similarly reported in a study done in Mozambique [52], Ghana [53], and India [54]. Mostly cereals were produced in the majority area and highly accessible in the market.

Vitamin A-rich fruits and vegetable consumption was 18% which is in line with a study done in Iran 19.98% [48]. Surely, those adolescents with low vitamin A consumption were at risk of other micronutrient deficiency [2]. On the other hand, vitamin A consumption among adolescent girls in this study was lower than a study conducted in Tigray 31.9% [50]. This difference could be variation in the study design and the presence of drought in the current study area which deteriorate cultivation of fruit and vegetables.

In the present study, milk consumption was 7.7% which is lower than the study conducted in India 37.25% [54], 79.2% in Iran [48], and 26.5% in Tigray Region [50]. This variation would be the socio-economic difference between the study participants and lack of animal source foods and water in the study area due to seasonal ELINO drought.

Being female was associated with inadequate dietary diversity. This finding is in line with a study done in Jimma [47] and contradicted to the study done in Iran [2] and Malaysia [55]. Females in Ethiopia are investing their times in food cooking; the smelling of the food might decrease the appetite. Practically women in Ethiopia spent more than 14 h in indoor and outdoor activity, which hampers their normal physiological need specifically daily dietary consumption [56]. Additionally females’ appetites are influenced by hormone secretion and female felt more satisfied easily.

The occurrence of inadequate dietary diversity decreased as the education of the mother increased; it was similarly reported in a study conducted in Nigeria [57] and South Africa [58]. It is true that the educational status of parents influences the dietary habit, food choice, and meal pattern of adolescents [17]. In fact, as the educational status of mothers increased, they have a chance to get information on healthy dietary habit. As a result, they formulate the dietary habit of the adolescent to be diversified [59]. Moreover, as mother’s educational level increases their income will grow and gives a chance to fulfill basic needs properly [56]. Generally, educated mothers can easily change nutrition knowledge to practice in food preparation because home activities and food preparation were covered by females most of the time in the Ethiopia context.

In this study, adolescents having poor knowledge of nutrition were positively associated with inadequate dietary diversity. The current study is supported by studies conducted in Luxembourg [60] and Jimma [47]. This is evidenced that as adolescents’ knowledge about disease increases, they start to take more diversified food [53]. Similarly, an evidence from Greece study revealed that having essential knowledge about food was important to maintain good health [61].

Inadequate dietary diversity among adolescents who live in family size of five and more was 2.05 times higher than adolescents living in family size less than five. The current finding is consistent with study conducted in South Africa [62] and Ethiopia [19]. It is a basic truth that as the number of family members increased, they face the economical insufficiency to meet their family needs. Because of this, they put their time to fulfill the daily needs, rather on diet quality.

Even though the study has several strengths, they had some little limitations. The limitation of this study was it did not address portion size estimation of food eaten by participants because of financial and time constraints. The data was collected through a 24-h dietary recall method which may be prone to recall and social desirability bias.

## Conclusion and recommendations

The proportion of inadequate dietary diversity was a significant figure which needs policy attention. Family size, sex, knowledge on nutrition, and education level of the mother were affecting the dietary diversity of adolescents. Animal source foods and fruit/vegetables were the least consumed food groups. In order to break intergenerational cycle of malnutrition, promoting diversified food consumption is a good opportunity, since today adolescents are tomorrow mothers Developing a nutrition education program and establishing a nutrition club in the school will be a better approach to reduce the problem. Moreover, formulating special nutrition education programs for mothers who have low educational status will improve the practice of diversified food consumption.

## Supplementary Information

**Additional file 1.** Peer Review Reports.

## Data Availability

The datasets used and/or analyzed during the current study are available from the corresponding author based on reasonable request.
