# Supplementary material for: Factors associated with dietary diversity among adolescents in Woldia, Northeast Ethiopia
Source: BMC Nutr. 2021 Apr 29;7:27. doi: 10.1186/s40795-021-00430-6 (PMC8082914; doi:10.1186/s40795-021-00430-6)
Supplement: Supplementary file 1 — Additional file 1. Peer Review Reports. [file 40795_2021_430_MOESM1_ESM.zip › ReviewerReport_V5_R2.pdf]

Title: Factors associated with dietary diversity among Adolescents in Woldia; Northeast Ethiopia

Version: 5 **Date:** 15 Dec 2020

Reviewer's report:

Thank you for this invitation. This manuscript examined dietary diversity and its related factors among adolescents in Woldia. The main finding was that The percentage of inadequate dietary diversity among study participants was 49.1 % (95 % CI: 44.5-53.8). inadequate dietary diversity was associated with sex, knowledge of nutrition, maternal education level, and family size.
There are several comments/suggestions that must improve this paper:
Overall, The English language require some improvement.
Some repetition needs to be addressed.
Abstract section should include the aim of the study and brief description of methods used (e.g. how dietary diversity was scored).
Methods section is not clearly explained and more details need to be included.
