# Supplementary material for: Factors associated with dietary diversity among adolescents in Woldia, Northeast Ethiopia
Source: BMC Nutr. 2021 Apr 29;7:27. doi: 10.1186/s40795-021-00430-6 (PMC8082914; doi:10.1186/s40795-021-00430-6)
Supplement: Supplementary file 1 — Additional file 1. Peer Review Reports. [file 40795_2021_430_MOESM1_ESM.zip › ReviewerReport_V8_R2.pdf]

Title: Factors associated with dietary diversity among Adolescents in Woldia; Northeast Ethiopia

Version: 8 **Date:** 27 Jan 2021

Reviewer's report:

no comments
