# Supplementary material for: Factors associated with dietary diversity among adolescents in Woldia, Northeast Ethiopia
Source: BMC Nutr. 2021 Apr 29;7:27. doi: 10.1186/s40795-021-00430-6 (PMC8082914; doi:10.1186/s40795-021-00430-6)
Supplement: Supplementary file 1 — Additional file 1. Peer Review Reports. [file 40795_2021_430_MOESM1_ESM.zip › AuthorComment_V6.pdf]

Cover letter to BMC Nutrition editors

General reflections to BMC Nutrition editors

Dear BMC Nutrition editors and reviewers, I hearty acknowledged your efforts made in each section of our manuscript. I am very happy with all the given comments and clarity questions that you raised. I know that the purpose of the review is improving the quality and readability of the manuscript. I fully accept them and did our possibilities by giving time to each section of a manuscript to address the given comment and clarity questions. My approaches were not focusing on answering only the raised issues, but revising each section by taking the raised issues as triggering points. **Frankly speaking**, I see this as an excellent opportunity to revise the manuscript and increase its quality.

Activities that I made are incorporating comments/suggestions, giving answers/explanations/ justifications/reasons/to clarity questions, adding concepts, inserting new words/phrases, deleting unnecessary words/phrases, improving language problems (type errors, wording, clarity, and sentence parallelism and complexity). Removing unnecessary spaces between words, sentences, paragraphs**…**are made. **The revisions are made to each section especially methods revised deeply.** I am sure that most of the clarity questions and comments given by the respected reviewers are addressed as much as possible.

Point by point response of Authors

**S.no**	**Reviewer 1**	**Authors response**
1.	Overall, The English language require some improvement. Some repetition needs to be addressed.	Dear reviewer, As per your comment the English language was edited intensively.
2.	Abstract section should include the aim of the study and brief description of methods used (e.g. how dietary diversity was scored).	It is revised as follows "So the aim of the study was to identify determinant factors of dietary diversity." A brief explanation on DDS "The out come variable was dietary diversity;it was calculated by summing of the number of food group consumed by individuals over a given period."
3.	Methods section is not clearly explained and more details need to be included.	Detailed revision was made and the change is accessed in tracked document.
4.	Reviewer 2	
5.	Dietary diversity was studied as a determinant factor for malnutrition	Revised intensively

	previously in adolescents, But the not capital letter	
6.	the description of the variables to create wealth index in the socio-econimic questionnaire is missing.	I saw the questionnaire ,it is already included in the questionnaire part four section
7.	what do you mean for 'recorded from hotel menus'? It is not clear	In Ethiopia context there is no food guide pyramid or any dietary guideline. The difficulty of conducting such study is unable to get list of foods. As a result in order to create the list of foods market observation is recommended. Because of this we are visit Hotels and we record the food item sold by them .So by visiting Many hotels in such method we gather a lot of food list .Then after compiling it ,we construct DDS questionnaire
8.	re-phrase the following sentence The questions nutrition knowledge questions are composed of the cause and prevention mechanism of (Iodine deficiency, Vitamin A deficiency, Iron deficiency anemia and malnutrition).	Thank you ,we revised it intensively.
9.	table 4 please check 0.284(0.1750.46)	Yes it is checked and corrected.
10.	a comment on the distribution of the percentage of adolescent of food groups between adequate and inadequate could be useful to better understand the DDS score and address the nutritional programs.	Appropriate reference was cited
11.	please add the reference for: Actually female in Ethiopia are invests their times through food cooking, the smelling of the food might decrease the appetite. Additionally females appetite are influenced by hormone secretion and female felt more satisfied easily	
12.	reference 6 : • Hoddinott, John & Yohannes, Yisehac, 2002. " and 7 are not complete check reference 39 and 46 and 47	All references was modified based on the comment given.

Thank you much. I accept your comment and revised all the documents and prepared based on journal standards.

Thank you much for your contributions!
