# Supplementary material for: Factors associated with dietary diversity among adolescents in Woldia, Northeast Ethiopia
Source: BMC Nutr. 2021 Apr 29;7:27. doi: 10.1186/s40795-021-00430-6 (PMC8082914; doi:10.1186/s40795-021-00430-6)
Supplement: Supplementary file 1 — Additional file 1. Peer Review Reports. [file 40795_2021_430_MOESM1_ESM.zip › ReviewerReport_V5_R1.pdf]

Title: Factors associated with dietary diversity among Adolescents in Woldia; Northeast Ethiopia

Version: 5 **Date:** 23 Dec 2020

Reviewer's report:

This study deal with the Factors associated with dietary diversity among Adolescents in a region of Ethiopia. It is very important the collection of this type of data because it is essential for planning nutritional interventions. The study design is well done and described. Congratulations
I have some minor comments:
Introduction
Dietary diversity was studied as a determinant factor for malnutrition previously in adolescents, But the
not capital letter
Data collection tools and procedure
the description of the variables to create wealth index in the socio-econimic questionnaire is missing.
what do you mean for 'recorded from hotel menus'? It is not clear

re-phrase the following sentence
The questions nutrition knowledge questions are composed of the cause and prevention mechanism of (Iodine deficiency, Vitamin A deficiency, Iron deficiency anemia and malnutrition).

table 4
please check 0.284(0.1750.46)
Discussion
a comment on the distribution of the percentage of adolescent of food groups between adequate and inadequate could be useful to better understand the DDS score and address the nutritional programs.
please add the reference for:
Actually female in Ethiopia are invests their times through food cooking, the smelling of the food might decrease the appetite. Additionally females appetite are influenced by hormone secretion and female felt more satisfied easily
References:
reference 6 and 7 are not complete
check reference 39 and 46 and 47
